# Prevalence and biopsychosocial associations of functional constipation: a cross-sectional study among adults in Türkiye

**DOI:** 10.1186/s12876-026-05062-3

**Published:** 2026-07-23

**Authors:** Cansu Memiç-İnan, Emre Duman, Eren Canbolat, Ayşe Nur Songür-Bozdağ, Mustafa Çapraz

**Affiliations:** 1 Department of Nutrition and Dietetics, Faculty of Health Sciences, Hitit University, Çorum, Türkiye; 2 Department of Nutrition and Dietetics, Faculty of Health Sciences, Siirt University, Siirt, Türkiye; 3 Department of Nutrition and Dietetics, Faculty of Health Sciences, Ankara University, Ankara, Türkiye; 4 Department of Nutrition and Dietetics, Faculty of Health Sciences, İzmir Katip Çelebi University, İzmir, Türkiye; 5 Department of Internal Medicine, Faculty of Medicine, Amasya University, Amasya, Türkiye

**Keywords:** Functional constipation, Eating disorder risk, Physical activity, Body mass index, Picky eating

## Abstract

**Background:**

Functional constipation is a prevalent functional gastrointestinal disorder with substantial public health implications. Evidence on its biopsychosocial determinants in middle-income settings is limited. This study aimed to determine the prevalence of functional constipation and its associations with sociodemographic characteristics, dietary habits, eating behaviors, physical activity, and anthropometric factors among adults in Türkiye.

**Methods:**

This study was conducted with 429 individuals aged 19–64 years who presented for examination and follow-up at the Department of Internal Medicine outpatient clinic of a public hospital in Türkiye. Functional constipation was defined by Rome IV criteria. Constipation severity was assessed with the Constipation Severity Instrument. Eating disorder risk was screened using the five-item The Sick, Control, One Stone, Fat, Food (SCOFF) Questionnaire; picky eating was measured with the Adult Picky Eating Questionnaire. Anthropometrics were measured to compute body mass index (BMI). Physical activity and selected dietary frequencies were self-reported. Predictors of functional constipation were examined via binary logistic regression with covariates informed by prior literature. Statistical significance was set at α = 0.05.

**Results:**

Participants’ mean age was 38.2 ± 15.0 years; 53.1% were female; 50.6% had BMI ≥ 25 kg/m². The prevalence of functional constipation was 21.0% (90/429). In multivariable analyses, higher age (OR = 1.029, 95% CI: 1.005–1.055) and female sex (OR = 1.899, 95% CI: 1.007–3.578) were associated with greater odds of functional constipation. Eating disorder risk was also associated with greater odds of functional constipation (per point on SCOFF: OR = 1.280, 95% CI: 1.024–1.600). In contrast, higher BMI was associated with lower odds (OR = 0.932, 95% CI: 0.879–0.989), and engaging in physical activity was associated with substantially lower odds of functional constipation (OR = 0.168, 95% CI: 0.095–0.300). Family history, smoking, alcohol use, water intake, and frequencies of meat, vegetable, and fruit consumption were not significant in the adjusted model; meal skipping showed borderline significance (OR = 2.001, 95% CI: 0.996–4.020; *p* = 0.051).

**Conclusions:**

The findings of the study indicate that functional constipation has a considerable prevalence in this clinical adult sample. Functional constipation was associated with older age, female sex, and eating disorder risk, and was inversely associated with higher BMI and physical activity. The results support the routine assessment of eating disorder risk and the promotion of physical activity as part of comprehensive management strategies. Prospective studies are needed to clarify the causal pathways underlying these observed associations.

## Introduction

Functional constipation (FC) is a common functional gastrointestinal disorder characterized by a decrease in bowel movement frequency, straining during defecation, and a sensation of incomplete evacuation, without an identifiable organic cause [1, 2]. Defined according to the Rome IV criteria, this condition not only affects individuals’ gastrointestinal health but also significantly impairs quality of life, psychological well-being, and social functioning, highlighting its importance as a public health issue [2, 3]. Epidemiological data indicate that the prevalence of FC varies widely worldwide, ranging from 0.2% to 30.7%, with reported rates of 8.5–16% in the adult population [3–5]. This epidemiological variability is also evident in regional studies; for instance, the prevalence in the general population in China is 8.5% [3], whereas it can reach up to 30% among the elderly population in Türkiye [6]. Such variation in prevalence reflects the multifactorial and complex nature of FC etiology.

The etiology of FC is based on a complex interaction of demographic, behavioral, psychological, and neurophysiological factors [7]. Advanced age is considered one of the strongest risk factors due to physiological changes such as decreased colonic motility and pelvic floor dysfunction. Similarly, female sex is more susceptible to the development of FC, likely due to hormonal differences and increased visceral sensitivity [3, 8–11].

In addition to physiological factors, individuals’ behavioral habits also play an important role in the development of FC. A sedentary lifestyle, in particular, can slow bowel motility and increase the risk of FC [3, 12]. Moreover, low dietary fiber intake and insufficient fluid consumption negatively affect stool volume and consistency, prolonging colonic transit time and contributing to the development of FC [6, 13]. In this context, eating behaviors that lead to low fiber and inadequate fluid intake may also trigger FC.

Picky eating behavior is defined as “the consumption of an insufficiently varied diet due to the rejection of both familiar and new foods” [14]. Beginning in childhood and potentially persisting into adulthood, picky eating behavior may lead to avoidance of fiber-rich vegetables, fruits, and legumes, resulting in chronically low fiber intake and thereby predisposing individuals to FC [14, 15]. Indeed, studies in children have reported an association between picky eating and clinical constipation or FC [16, 17]. However, the relationship between picky eating and FC has not yet been evaluated in adults.

The relationship between eating disorders, a more severe form of disrupted eating behaviors, and FC is also noteworthy. Factors such as energy restriction, electrolyte imbalances, and laxative misuse observed in conditions like anorexia nervosa and bulimia nervosa can impair gastrointestinal motility, thereby increasing the risk of FC [18–21]. Indeed, it has been reported that approximately 95% of individuals diagnosed with eating disorders exhibit functional gastrointestinal symptoms, with FC (35–40%) being among the most frequently observed [22]. Furthermore, in recent years, the role of psychosocial factors affecting FC pathophysiology through the brain-gut axis has been increasingly discussed [23–26].

However, the effects of certain lifestyle factors on FC have not yet been fully elucidated. For example, the relationship between body mass index (BMI) and constipation is inconsistent; some studies identify high BMI as a risk factor [27], while others report higher FC prevalence in individuals with normal or low BMI [3]. Similarly, although it has been suggested that smoking might have a potential protective role due to nicotine’s stimulatory effect on colonic motility [28], or that alcohol consumption might be inversely associated with constipation [29], the underlying mechanisms of these relationships remain unclear.

Taken together, these findings indicate that FC is not merely a mechanical bowel problem but rather a complex biopsychosocial disorder involving intertwined biological, psychological, and behavioral components. This complex etiological background underscores the need for a holistic approach in the prevention and management of FC. In this context, the present study primarily aimed to comprehensively examine the associations of modifiable factors—such as dietary habits and behaviors, physical activity level, and BMI—alongside sociodemographic characteristics with FC. By doing so, it seeks to provide a scientific basis for the development of more comprehensive and sustainable strategies for the prevention and management of FC.

## Methods

### Participants

The sample for this cross-sectional study consisted of individuals aged 19–64 years who applied for examination and follow-up at the Department of Internal Medicine, Amasya University Sabuncuoğlu Şerefeddin Training and Research Hospital. Data were collected between January and April 2025. In determining the sample size, relevant literature [30, 31] was reviewed, and using G*Power software, a minimum of 420 participants was calculated to be necessary based on an alpha level (α) of 0.05, a power (1 − β) of 0.80, and the specified effect size. The inclusion criteria for the study are presented below.

1. Aged between 19 and 64 years.
2. Not pregnant or breastfeeding.
3. Without medical conditions such as irritable bowel syndrome (IBS), celiac disease, colorectal cancer, intestinal mass, autonomic neuropathy, or food allergies.
4. Not using medications or supplements such as laxatives, opioids, calcium channel blockers, antidepressants, pre/probiotics, or magnesium supplements.
5. Had not made any dietary changes in the past month.
6. Provided written informed consent.

**Fig. 1:**
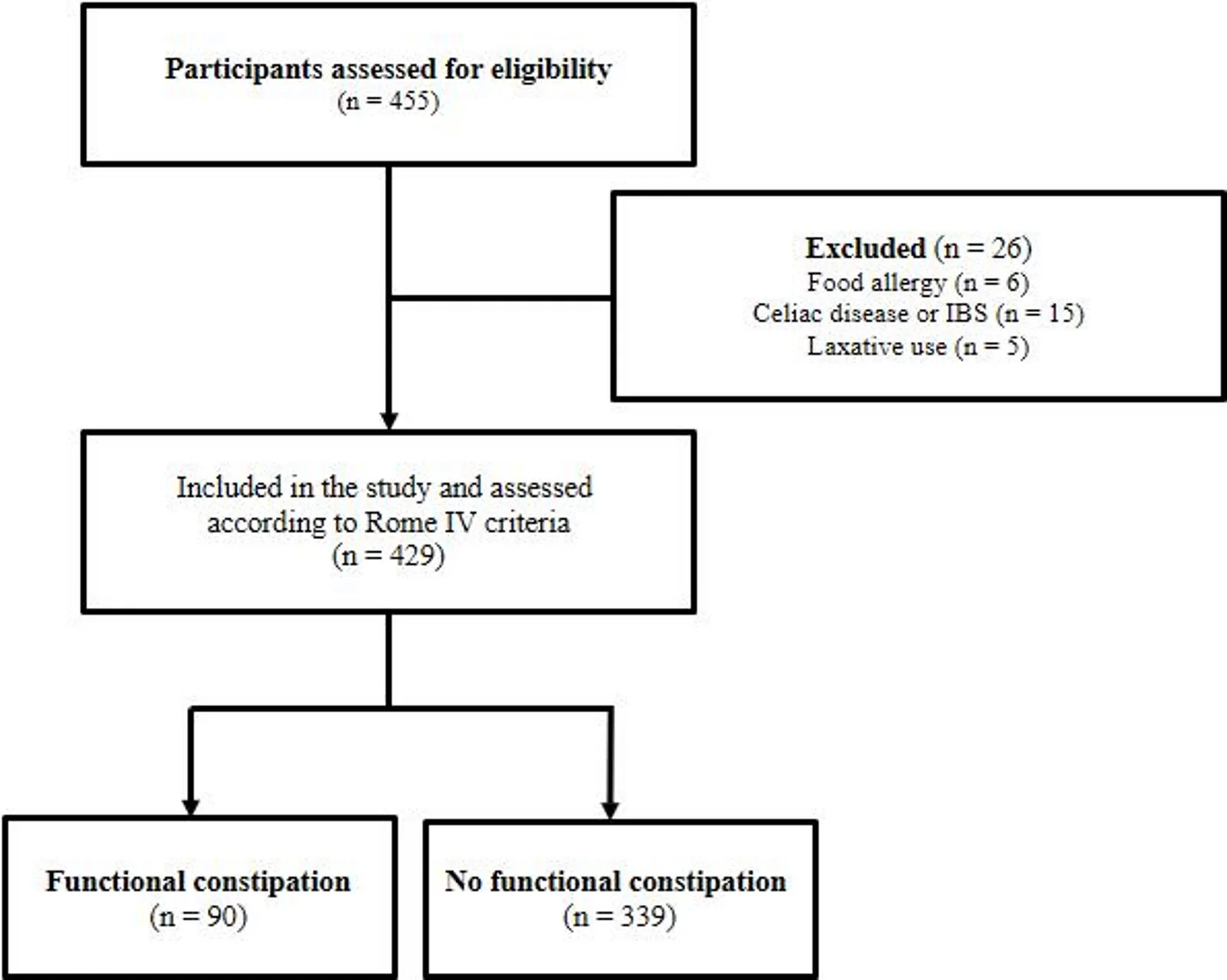
Participant flow diagram (IBS: Irritable bowel syndrome)

A total of 455 individuals were reached for the study. However, some were excluded for various reasons: those with food allergies (*n* = 6), diagnosed with celiac disease or IBS (*n* = 15), and those using laxatives (*n* = 5). Ultimately, 429 adult individuals comprised the study sample. A flow diagram illustrating participant selection and exclusion is presented in Fig. 1. Ethical approval for the study was obtained from the Amasya University Ethics Committee (No. 2024/93). The study was conducted in accordance with the Declaration of Helsinki, and all participants provided voluntary informed consent. Reporting of the study followed the STROBE (Strengthening the Reporting of Observational Studies in Epidemiology) guidelines.

### Study design

In this study, participants completed a survey through face-to-face interviews. The survey used to collect study data included sections on participants’ general information, questions to determine FC status, the Constipation Severity Instrument (CSI), the The Sick, Control, One, Fat, Food (SCOFF) Questionnaire, and the Adult Picky Eating Questionnaire (APEQ).

### Participants’ general information

Information on participants’ sex, age, family history of constipation, smoking and alcohol consumption, physical activity status, and certain dietary habits was collected and recorded in the survey form. Participants were asked, “How often do you consume meat (chicken, fish, red meat), fruits, and vegetables?” Responses were initially categorized into five options: daily, 5–6 days per week, 3–4 days per week, 1–2 days per week, and never. For analysis, these responses were collapsed into three categories based on sample distribution: frequent consumption (daily and 5–6 days per week), moderate consumption (3–4 days per week), and infrequent or no consumption (1–2 days per week or never).

Additionally, participants’ body weight (kg) and height (cm) were measured. A calibrated scale with 0.1 kg sensitivity was used for weight measurement. Participants were measured while fasting, with heavy clothing and shoes removed. Height was measured using a stadiometer with participants in the Frankfurt plane, feet together, and the head, hips, and heels aligned parallel to the wall, with shoes removed [32]. BMI was calculated by dividing body weight (kg) by the square of height (m²). Participants with a BMI below 18.5 were classified as underweight, 18.5–24.99 as normal weight, 25.0–29.99 as overweight, and 30.0 or higher as obese [33]. In this study, BMI was evaluated in two categories: underweight and normal weight (BMI < 25 kg/m²) and overweight and obese (BMI ≥ 25 kg/m²).

### Definition of functional constipation

The diagnosis of FC was made according to the Rome IV criteria. According to these criteria, the presence of all three conditions (I, II, and III) is required for an FC diagnosis. Additionally, symptoms must have started at least six months prior to diagnosis and persisted during the last three months [34].

1. Presence of two or more of the following symptoms:
  - Straining during more than 25% of bowel movements.
  - Lumpy or hard stools in more than 25% of bowel movements.
  - Sensation of incomplete evacuation in more than 25% of bowel movements.
  - Sensation of obstruction or blockage in the anorectal region in more than 25% of bowel movements.
  - Manual maneuvers to facilitate defecation in more than 25% of bowel movements.

2. Rare occurrence of soft, shapeless stools without the use of laxatives.
3. Insufficient criteria for a diagnosis of irritable bowel syndrome (IBS).

### The Constipation Severity Instrument (CSI)

The Constipation Severity Instrument (CSI), developed by Varma et al. [35] and validated in Turkish by Kaya and Turan [36], is used to assess individuals’ bowel movement frequency, intensity, and difficulty during defecation. The scale consists of 16 items and includes three subscales: Obstructive Defecation, Colonic Inertia, and Pain. Scores for the OD subscale range from 0 to 28, for the CI subscale from 0 to 29, and for the Pain subscale from 0 to 16. Total scores on the scale range from 0 to 73, with higher scores indicating more severe symptoms. Kaya and Turan [36] reported a Cronbach’s α value of 0.92 for the scale.

### The Sick, Control, One stone, Fat, Food (SCOFF) questionnaire

The Sick, Control, One Stone, Fat, Food Questionnaire, developed by Hill et al. [37], was used to screen for the risk of eating disorders. The scale was adapted into Turkish by Aydemir et al. [38]. The questionnaire consists of 5 items, and during evaluation, each “yes” response is scored as one point. Total scores range from 0 to 5, with a score of 2 or higher indicating a risk for an eating disorder. The scale’s internal consistency, as measured by Cronbach’s alpha, has been reported as 0.74.

### The Adult Picky Eating Questionnaire (APEQ)

The Adult Picky Eating Questionnaire (APEQ), developed by Ellis et al. [39] to assess picky eating in adults, was used in this study. The Turkish validity and reliability of the scale were established by Ayyıldız and Esin [40]. The original APEQ consisted of 16 items; however, in the Turkish validation, two items with low factor loadings (items 2 and 3) were removed, resulting in a 14-item Turkish version [39]. The scale uses a five-point Likert format, with scores ranging from 1 = “Never” to 5 = “Always.” The total score is calculated by summing the scores of all items and dividing by the number of items. Higher scores indicate a greater tendency toward picky eating.

### Statistical analyses

Data obtained from the study were analyzed using Statistical Package for the Social Sciences (SPSS) version 26.0. The normality of variables was assessed using both visual (histogram, Q–Q plot) and analytical (Kolmogorov–Smirnov test) methods. Descriptive data were presented as numbers and percentages. Analyses showed that the data were normally distributed. Differences between two groups were evaluated using the independent samples t-test. For categorical variables, Fisher’s exact test was applied when more than 20% of expected cell counts were less than 5 or any expected cell count was less than 1; otherwise, Pearson’s chi-square test was used. Correlations between normally distributed continuous variables were determined using the Pearson correlation test. Binary logistic regression analysis was performed to identify factors affecting the presence of FC. Covariates were selected based on the literature and the results of the preliminary analyses. In addition to variables showing associations with the dependent variable in the preliminary analyses, variables reported in the literature to be associated with functional constipation or considered clinically important were also included in the multivariable logistic regression model, even if they did not show statistically significant associations. For descriptive purposes, some variables (e.g., age and BMI) were presented in categorized form; however, to avoid loss of information, these variables were entered into the logistic regression model as continuous variables. All analyses were evaluated at a significance level of 0.05, and p-values for statistically significant results are presented in bold.

## Results

The characteristics of the participants are presented in Table 1. Of the participants, 34.0% were aged 46–64 years (mean age 39.3 ± 14. years), 53.1% were female, and 50.6% were overweight or obese (mean BMI 25.1 ± 4.2 kg/m²). Individuals with a family history of chronic constipation comprised 32.4% of the sample. Among participants, 40.6% reported smoking and 19.8% reported alcohol consumption. The frequency of participants who reported being physically active was 64.8%, while 73.2% reported skipping meals. According to the Rome IV criteria, 21.0% of participants had FC. The mean total constipation severity score was 18.4 ± 10.6, with subscale mean scores of 9.0 ± 5.7 for obstructive defecation, 8.6 ± 5.0 for colonic inertia, and 0.9 ± 1.8 for pain. The mean APEQ score was 2.5 ± 0.6, and the mean SCOFF score was 1.5 ± 1.3.

**Table 1:** Characteristics of participants

Characteristic	Numbers (%)
	Mean ± SD
Age (years)	39.3 ± 14.3
19–29	144 (33.6)
30–45	139 (32.4)
46–64	146 (34.0)
Sex	
Male	201 (46.9)
Female	228 (53.1)
BMI (kg/m^2^ )	25.1 **±** 4.2
< 25 kg/m^2^ (underweight and normal weight)	212 (49.4)
≥ 25 kg/m^2^ (overweight and obese)	217 (50.6)
Family history of chronic constipation	
Yes	139 (32.4)
No	290 (67.6)
Smoking	
Yes	174 (40.6)
No	255 (59.4)
Alcohol	
Yes	85 (19.8)
No	344 (80.2)
Physical activity	
Yes	278 (64.8)
No	151 (35.2)
Meal skipping	
Yes	314 (73.2)
No	115 (26.8)
Functional constipation	
Yes	90 (21.0)
No	339 (79.0)
CSI score	18.4 ± 10.6
OD score	9.0 ± 5.7
CI score	8.6 ± 5.0
Pain score	0.9 ± 1.8
APEQ score	2.5 ± 0.6
SCOFF score	1.5 ± 1.3

Note: Age categorized by 33rd and 66th percentiles

*BMI* Body Mass Index, *APEQ* Adult Picky Eating Questionnaire, *SCOFF* Sick, Control, One Stone, Fat, Food, *CSI* Constipation Severity Instrument, *OD* Obstructive Defecation, *CI* Colonic Inertia

Table 2 presents the general characteristics and dietary habits of participants according to FC status. Age, sex, smoking and alcohol consumption, physical activity status, frequency of vegetable and meat consumption, water intake, and SCOFF scores differed significantly according to FC status (*p* < 0.05). Among individuals with FC, a higher proportion were aged 31–45 years, female, non-smokers, non-drinkers, physically inactive, and consumed meat less frequently (*p* < 0.05). Additionally, participants with FC had higher mean age and SCOFF scores, while their daily water intake was lower (*p* < 0.05). The proportion of individuals who frequently consumed vegetables was higher among those without FC (*p* < 0.05).

**Table 2:** General characteristics and dietary habits of participants according to functional constipation status

	No functional constipation	Functional constipation	*p*
Age (years)	38.5 ± 14.9	42.3 ± 11.0	**0.008**
19–29	130 (38.3)	14 (15.6)	**< 0.001**
30–45	95 (28.0)	44 (48.9)	
46–64	114 (33.6)	32 (35.6)	
Sex			
Male	175 (51.6)	26 (28.9)	< **0.001**
Female	164 (48.4)	64 (71.1)	
BMI			
< 25 kg/m^2^	167 (49.3)	45 (50.5)	0.901
≥ 25 kg/m^2^	172 (50.7)	45 (50.0)	
Family history of chronic constipation			
Yes	110 (32.4)	29 (32.2)	0.967
No	229 (67.6)	61 (67.8)	
Smoking			
Yes	148 (43.7)	26 (28.9)	**0.011**
No	191 (56.3)	64 (71.1)	
Alcohol			
Yes	77 (22.7)	8 (8.9)	**0.003**
No	262 (77.3)	82 (91.1)	
Physical activity			
Yes	250 (73.7)	28 (31.1)	**< 0.001**
No	89 (26.3)	62 (68.9)	
Meal skipping			
Yes	242 (71.4)	72 (80.0)	0.101
No	97 (28.6)	18 (20.0)	
Frequency of fruit consumption			
Frequent	136 (40.1)	34 (37.8)	0.541
Moderate	99 (29.2)	23 (25.6)	
Rare/Never	104 (30.7)	33 (36.7)	
Frequency of vegetable consumption			
Frequent	160 (47.2)	35 (38.9)	**0.035**
Moderate	122 (36.0)	29 (32.2)	
Rare/Never	57 (16.8)	26 (28.9)	
Frequency of meat consumption			
Frequent	68 (20.1)	11 (12.2)	**0.045**
Moderate	130 (38.3)	29 (32.2)	
Rare/Never	141 (41.6)	50 (55.6)	
Water intake (ml/day)	2137.0 ± 864.7	1871.1 ± 820.4	**0.009**
APEQ score	2.5 ± 0.6	2.6 ± 0.6	0.114
SCOFF score	1.4 ± 1.2	1.8 ± 1.3	**0.007**

The *p* values that are statistically significant (*p* < 0.05) should be presented in bold

*BMI* Body Mass Index, *APEQ* Adult Picky Eating Questionnaire, *SCOFF* Sick, Control, One Stone, Fat, Food

**Fig. 2:**
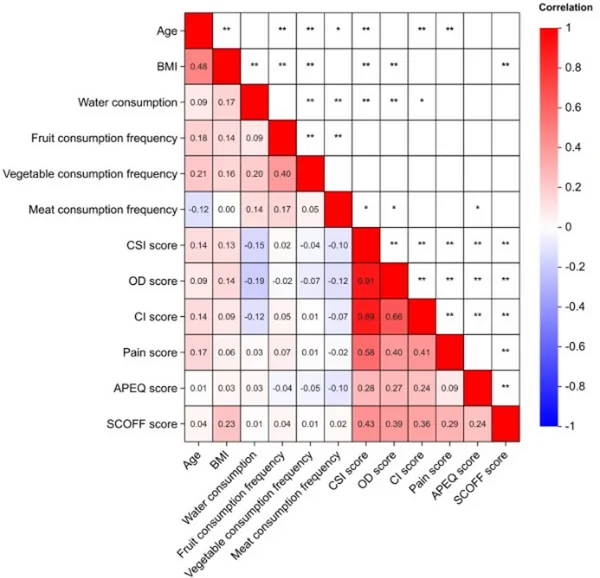
Correlations among study variables (BMI: Body Mass Index; APEQ: Adult Picky Eating Questionnaire; SCOFF: Sick, Control, One Stone, Fat, Food; CSI: Constipation Severity Instrument; OD: Obstructive Defecation; CI: Colonic Inertia. * < 0.05, ** < 0.001)

Figure 2 presents the correlations among the study variables. The CSI score was found to be positively correlated with the SCOFF score (*r* = 0.43), APEQ score (*r* = 0.28), age (*r* = 0.14), and BMI (*r* = 0.13), and negatively correlated with water intake (*r* = -0.15) and frequency of meat consumption (*r* = -0.10) (*p* < 0.05). Additionally, the CSI subscales—obstructive defecation, colonic inertia, and pain—were also significantly positively correlated with SCOFF and APEQ scores (*p* < 0.05).

Table 3 presents the results of the binary logistic regression analysis examining factors associated with FC. According to the Hosmer–Lemeshow goodness-of-fit test, the model demonstrated an acceptable fit to the data (χ² = 13.096, *p* = 0.109). The Nagelkerke R² value was 0.304, indicating that the model explained approximately 30.4% of the variance in FC. The analysis showed that age, female sex, SCOFF score, BMI, and physical activity were significantly associated with FC. Increasing age was associated with greater odds of FC (OR = 1.029, 95% CI: 1.005–1.055), and females had greater odds of FC compared to males (OR = 1.899, 95% CI: 1.007–3.578). The SCOFF score also was significantly associated with greater odds of FC (OR = 1.280, 95% CI: 1.024–1.600). In contrast, higher BMI was associated with lower odds of FC (OR = 0.932, 95% CI: 0.879–0.989). Individuals engaging in physical activity had significantly lower odds of FC compared to those who did not (OR = 0.168, 95% CI: 0.095–0.300). Other variables, including APEQ score, family history of chronic constipation, smoking, alcohol consumption, water intake, and frequency of meat, vegetable, and fruit consumption, were not statistically significant factors associated with FC (*p* > 0.05). Skipping meals showed borderline significance (OR = 2.001, 95% CI: 0.996–4.020, *p* = 0.051).

**Table 3:** Predictors of Functional Constipation (Binary Logistic Regression Analysis)

Variable	Odds Ratio	95% CI for odds ratio		*p*
		Lower	Upper	
SCOFF score	1.280	1.024	1.600	**0.030**
APEQ score	0.999	0.967	1.031	0.935
Age (years)	1.029	1.005	1.055	**0.020**
Sex				
Male (Ref)	-	-	-	-
Female	1.899	1.007	3.578	**0.047**
BMI (kg/m^2^)	0.932	0.879	0.989	**0.020**
Physical activity				
No (Ref)	-	-	-	-
Yes	0.168	0.095	0.300	**< 0.001**
Family history of chronic constipation				
No (Ref)	-	-	-	-
Yes	0.917	0.477	1.763	0.794
Smoking				
No (Ref)	-	-	-	-
Yes	0.591	0.310	1.126	0.110
Alcohol				
No (Ref)	-	-	-	-
Yes	0.553	0.220	1.392	0.209
Skipping meals				
No (Ref)	-	-	-	-
Yes	2.001	0.996	4.020	0.051
Water intake (mL/day)	1.000	0.999	1.000	0.449
Meat consumption frequency				
Rare/Never (Ref)	-	-	-	-
Moderate	1.030	0.551	1.928	0.926
Frequent	0.844	0.362	1.965	0.693
Vegetable consumption frequency				
Rare/Never (Ref)	-	-	-	-
Moderate	0.564	0.269	1.181	0.129
Frequent	0.519	0.242	1.113	0.092
Fruit consumption frequency				
Rare/Never (Ref)	-	-	-	-
Moderate	0.922	0.449	1.895	0.825
Frequent	1.029	0.497	2.128	0.939

*BMI *Body Mass Index, *APEQ* Adult Picky Eating Questionnaire, *SCOFF* Sick, Control, One Stone, Fat, Food, *Ref* Reference, *CI* Confidence Interval, *OR* Odds Ratio

## Discussion

This study examined the prevalence and multidimensional factors associated with FC in adults based on the current diagnostic standard, the Rome IV criteria, and revealed findings of both epidemiological and clinical significance. The FC prevalence of 21.0% observed in our study further underscores the public health importance of this syndrome. Although Rome IV criteria generally yield lower prevalence estimates than previous diagnostic frameworks [4, 41, 42], the prevalence observed in our study was somewhat higher than that reported in large population-based studies using Rome IV criteria [4, 43]. This difference may be explained by the clinical nature of our sample, which consisted of individuals attending an internal medicine outpatient clinic rather than a community-based population. Therefore, our findings support the considerable burden of FC in healthcare-seeking adult populations.

Our multivariable logistic regression analysis suggested that FC may represent a multidimensional condition shaped by biological, demographic, behavioral, and psychosocial factors. In our analysis, advanced age (OR = 1.029; 95% CI: 1.005–1.055; *p* = 0.020) and female sex (OR = 1.899; 95% CI: 1.007–3.578; *p* = 0.047) were independently associated with FC. The increased risk with age can be explained by factors such as slowed colonic motility, increased medication use, and accumulation of comorbid conditions [2, 44, 45]. The sex difference is striking, with FC prevalence in female being more than twice that in male (Table 2). This is closely related to the sensitivity of gastrointestinal motility to hormonal factors in female. Specifically, hormonal changes during the luteal phase of the menstrual cycle (estrogen/progesterone) and fluctuations in estrogen during menopause have been reported to slow colon transit time [13, 45]. In addition, changes in pelvic floor anatomy during pregnancy and postpartum, the higher prevalence of comorbid irritable bowel syndrome in female, and sex-related differences in symptom reporting and healthcare-seeking behaviors may also be additional social-psychological factors explaining this marked prevalence difference [10, 47, 48].

One of the most striking findings of our study is the significant inverse relationship between BMI and FC (OR = 0.932; 95% CI: 0.879–0.989; *p* = 0.020) (Table 3). At first glance, this finding may appear to contradict studies in the literature reporting obesity as a risk factor [27, 49]; however, it can be explained by hypotheses suggesting that this relationship may not be linear and could follow a U-shaped curve [50]. Moreover, there are studies in the literature consistent with our findings that report an association between low BMI and the prevalence of constipation. For example, Ogasawara et al. [51], in a large-scale online survey conducted in Japan, found that male with constipation had significantly lower BMI. Therefore, the association between BMI and FC may vary across diagnostic and symptom burden dimensions, which is also compatible with cross-sectional designs and different measurement frameworks. It should also be noted that low BMI may be closely related to SCOFF scores—identified as significantly associated with FC in our study (Fig. 2)—and may function as a somatic manifestation of restrictive eating behaviors.

The identification of eating disorder risk as an independent factor associated with FC (OR = 1.280; 95% CI: 1.024–1.600; *p* = 0.030) (Table 3) suggests that the association between eating disorder risk and FC may involve mechanisms that warrant further investigation. This finding highlights the necessity of understanding FC not merely as a mechanical problem but within a holistic model that includes behavioral and emotional dimensions [22]. Indeed, Huang et al. [23], in a large-scale study of U.S. adults, reported that constipation was independently associated with anxiety. Behavioral patterns commonly observed in individuals at risk for eating disorders—such as irregular meal skipping, highly restrictive diets, and diets often deficient in fiber—may directly increase the risk of constipation [52]. Beyond this behavioral dimension, disruptions in neurohormonal pathways, including hormones related to appetite and stress such as ghrelin, leptin, and corticotropin-releasing hormone (CRH), have also been shown to adversely affect gut motility and secretory functions [53–55].

Another noteworthy finding of this study was the relationship between constipation symptom severity and eating-related behaviors. CSI scores showed positive correlations with SCOFF and APEQ scores (Fig. 2), although the strength of these associations ranged from weak to moderate. These findings suggest that individuals with greater eating disorder risk and more pronounced picky eating tendencies may experience a higher burden of constipation symptoms. Murray et al. [56] reported a positive association between the severity of eating disorder pathology and constipation symptom severity. Furthermore, eating disorder psychopathology has been shown to predict the persistence and severity of constipation symptoms in individuals with anorexia nervosa [20]. Although the cross-sectional design precludes causal inference, these findings support the notion that behavioral and psychosocial factors may influence not only the presence of FC but also its clinical severity.

Physical inactivity was identified in our study as one of the factors most strongly associated with FC, whereas regular physical activity was associated with lower odds of FC (OR = 0.168; 95% CI: 0.095–0.300; *p* < 0.001) (Table 3). This strong association can be explained by both biological and behavioral mechanisms. Regular physical activity mechanically shortens colonic transit time, increases bowel movement frequency, and improves its function [2, 57]. This finding is consistent with studies reporting that physical inactivity increases the risk of FC [45, 58] and with observations noting increased constipation symptoms during the COVID-19 pandemic due to a more sedentary lifestyle [59].

The consumption indicators used in our study (frequency of fruit, vegetable, and meat intake) did not show a significant association in the multivariable model (*p* > 0.05) (Table 3). However, this finding likely reflects methodological limitations of the measurement tools rather than the true absence of dietary effects. Self-reported food frequency questionnaires generally cannot measure portion sizes, preparation methods, and, most importantly, total daily dietary fiber intake (in grams) with sufficient accuracy [60–62]. Furthermore, other dietary factors known to influence constipation, such as dairy products, coffee consumption, and ultra-processed foods, were not assessed in the present study. The omission of these dietary components may have limited our ability to comprehensively evaluate the relationship between dietary habits and FC. In addition, the presence of strong covariates in the statistical model, such as physical activity and SCOFF scores, may have statistically overshadowed or masked the effects of weaker signals like dietary intake. Indeed, increasing fiber consumption has been consistently reported to reduce FC symptoms [13, 63]. Therefore, it is more appropriate to interpret these negative findings in our study within the context of methodological limitations rather than as evidence of a true null effect.

In our study, although significant differences in smoking and alcohol consumption were observed between participants with and without FC, neither variable remained significantly associated with FC in the multivariable model. This discrepancy may reflect the influence of confounding factors. Additionally, reverse causation, reporting bias, and cultural differences in smoking and alcohol consumption behaviors may have contributed to these findings. Therefore, these associations should be interpreted with caution.

### Limitations of the study

The primary limitation of this study stems from its cross-sectional design; while this design allows for the identification of associations between variables, it does not permit conclusions regarding causality. For example, it cannot be determined whether a high SCOFF score causes constipation or whether the discomfort caused by chronic constipation adversely affects eating behaviors. Other limitations include potential recall bias and social desirability effects due to self-reported data, limited generalizability of findings because the sample was drawn from a single center, and, as detailed above, quantitative shortcomings in dietary assessment. Physical activity was assessed only as a dichotomous variable (yes/no), preventing a more detailed evaluation of exercise frequency, duration, and intensity. In addition, selection bias, measurement bias, and residual confounding cannot be completely excluded and should be considered when interpreting the findings.

## Conclusion

In conclusion, our study demonstrates that FC is a significant public health concern and is associated with multiple interrelated factors, including age, sex, physical activity, BMI, and eating behaviors. Our findings support the validity of a biopsychosocial model and strongly emphasize the inadequacy of standardized, one-size-fits-all approaches in clinical practice, highlighting the need for personalized and multifaceted interventions. Clinicians evaluating patients presenting with FC symptoms should systematically address not only the symptoms themselves but also physical activity levels, eating behaviors (e.g., using simple screening tools such as SCOFF), and psychosocial status. In female patients, careful monitoring of menstrual and menopausal periods may facilitate better management of symptom fluctuations. Behavioral interventions, particularly increasing physical activity and reducing sedentary behaviors, should be central to treatment plans. In cases accompanied by dyspepsia, anxiety, or eating disorders, multidisciplinary approaches involving gastroenterology, psychiatry, and nutrition specialists are likely to yield more effective outcomes.

Future research should focus on addressing the limitations of this study. Longitudinal (prospective) designs are strongly needed to better understand causal pathways. For more objective measurement of behavioral factors, wearable technologies such as accelerometers (for physical activity) and 24-hour dietary recall records (to quantify total fiber intake in grams per day) should be employed. To investigate the role of the gut-brain axis more thoroughly, measurement of neurohormonal markers (e.g., ghrelin, leptin) in blood is recommended. Finally, to clarify the complexity of the BMI–FC relationship, nonlinear modeling techniques (e.g., restricted cubic spline analyses) may be utilized. Such comprehensive approaches will enhance understanding of the causal pathways of FC and provide more robust evidence to inform clinical decision-making.

## Data Availability

The datasets are available from the corresponding author on reasonable request.

## Abbreviations

APEQ: Adult picky eating questionnaire
BMI: Body mass index
CI: Colonic inertia
CSI: Constipation severity instrument
FC: Functional constipation
IBS: Irritable bowel syndrome
OD: Obstructive defecation
Ref: Reference
SCOFF: Sick, control, one stone, fat, food
SPSS: Statistical package for the social sciences
